# Isolation, Characterization, and Application of Bacteriophage LPSE1 Against *Salmonella enterica* in Ready to Eat (RTE) Foods

**DOI:** 10.3389/fmicb.2018.01046

**Published:** 2018-05-23

**Authors:** Chenxi Huang, Safiullah M. Virk, Jianchun Shi, Yang Zhou, Stephan P. Willias, Mohamed K. Morsy, Hazem E. Abdelnabby, Jie Liu, Xiaohong Wang, Jinquan Li

**Affiliations:** ^1^Bio-Medical Center, Key Laboratory of Environment Correlative Dietology, State Key Laboratory of Agricultural Microbiology, College of Food Science and Technology, Huazhong Agricultural University, Wuhan, China; ^2^College of Fisheries, Huazhong Agricultural University, Wuhan, China; ^3^Department of Infectious Diseases and Pathology, University of Florida, Gainesville, FL, United States; ^4^Department of Food Technology, Faculty of Agriculture, Benha University, Benha, Egypt; ^5^College of Medicine, Hebei University of Engineering, Handan, China

**Keywords:** bacteriophage, LPSE1, *Salmonella*, milk, sausage, lettuce

## Abstract

*Salmonella* infection is an important foodborne consumer health concern that can be mitigated during food processing. Bacteriophage therapy imparts many advantages over conventional chemical preservatives including pathogen specificity, natural derivation, potency, and providing a high degree of safety. The objective of this study aimed to isolate and characterize a phage that effectively control *Salmonella* food contamination. Out of 35 isolated phages, LPSE1 demonstrated a broad *Salmonella* host range, robust lytic ability, extensive pH tolerance, and prolonged thermal stability. The capacity for phage LPSE1 to control *Salmonella* Enteritidis-ATCC13076 in milk, sausage, and lettuce was established. Incubation of LPSE1 at 28°C in milk reduced recoverable *Salmonella* by approximately 1.44 log_10_ CFU/mL and 2.37 log_10_ CFU/mL at MOI of 1 and 100, respectively, as relative to the phage-excluded control. Upon administration of LPSE1 at an MOI of 1 in sausage, *Salmonella* count decreased 0.52 log_10_ at 28°C. At MOI of 100, the count decreased 0.49 log_10_ at 4°C. Incubation of LPSE1 on lettuce reduced recoverable *Salmonella* by 2.02 log_10,_ 1.71 log_10_, and 1.45 log_10_ CFU/mL at an MOI of 1, 10, and 100, respectively, as relative to the negative control. Taken together, these findings establish LPSE1 as an effective weapon against human pathogenic *Salmonella* in various ready to eat foods.

## Introduction

*Salmonella enterica* is one of the biggest threats to human health which can cause significant economic losses in terms of medication and other expenditures (WHO, 2017). Within the United States alone, there are approximately 48 million annual cases of foodborne illness resulting in 128,000 hospitalizations and 3,000 deaths (CDC, 2016; Scallan et al., 2011). *Salmonella* is the most common cause of the acquired bacterial foodborne illness named as Salmonellosis. This disease is characterized by fever, diarrhea and other symptoms (Pui et al., 2011). Almost all strains of *Salmonella* are pathogenic and are predominately harbored in eggs, meats, animal products such as milk, or contaminated vegetables causing disease in human beings consuming the contaminated food The pathogen load in foods are predominantly controlled by use of special preservatives (Juneja et al., 2012) and also heat treatment in liquid foods. However, the risks of adverse side effects conferred by chemical preservatives are deterring (Pawlowska et al., 2012). Secondly, heat treatment can cause degradation of nutrients. It can also produce health threatening advanced glycation end products (Uribarri et al., 2010) and introduce some unwanted flavors in foods. Furthermore, the use of antibiotics in food products is largely discouraged due to long-term environmental stability and non-specific antimicrobial activity. To the contrary, because of bacteriophages’ habit of being obligate, these are attractive antimicrobial agents due to targeted pathogen host specificity, rapid killing, and self-replicating ability. In addition to obligate bacteriophages some generalized phages are also present keeping within the boundaries of bacterial specificity (Flores et al., 2011) proving bacteriophages do not harm eukaryotic cells and afford a high degree of safety (McCallin et al., 2013).

The first reported use of bacteriophages is dated back to early 20th century (García et al., 2008). At that time phages were used to control various disease including cholera, dysentery and also some diseases caused by *Salmonella* (Summers, 2012). In the present era of antibiotics and chemical treatments some bacteriophages are being used in a variety of foods to control *Salmonella*, *E. coli* O157:H7, *Listeria monocytogenes* in various ready to eat (RTE) and processed foods (Abuladze et al., 2008; Albino et al., 2014; Atterbury et al., 2007; Bao et al., 2015; Bardina et al., 2012; Bielke et al., 2007; Carter et al., 2012; Galarce et al., 2016; Hungaro et al., 2013; Kang et al., 2013; O’Flynn et al., 2006; Sparvero et al., 2009; Spricigo et al., 2013). Keeping in view the great efficacy of bacteriophages in controlling pathogens, the present study aims to isolate and characterize bacteriophages which effectively target human pathogenic *Salmonella*. Moreover, we seek to establish the potential of candidate phages to control *Salmonella* contamination in ready to eat (RTE) foods including milk, sausage, and lettuce.

## Materials and Methods

### Strains

*Salmonella enterica* serovar Enteritidis ATCC 13076 was used to isolate bacteriophages. *Salmonella enterica* serovar Enteritidis ATCC 13076, *Salmonella enterica* serovar Typhimurium ATCC 14028, *Salmonella enterica* serovar Typhimurium ATCC 13311, *Salmonella enterica* serovar Anatum ATCC 9270, *Salmonella enterica* serovar Choleraesuis ATCC 10708, *Listeria monocytogenes* ATCC 19114, *Vibrio parahaemolyticus* ATCC 33846, *Staphylococcus aureus* ATCC 29213, *Staphylococcus aureus* ATCC 6538 [American Type Culture Collection (ATCC)]; *Salmonella enterica* serovar Enteritidis SJTUF 10978 and *Salmonella enterica* serovar Enteritidis SJTUF 10984 [Shanghai Jiao Tong University (SJTU)]; *Salmonella enterica* serovar Paratyphi B CMCC 50094 [National Center for Medical Culture Collection (CMCC)]; *Salmonella enterica* serovar Pullorum CVCC 534 [China Veterinary Culture Collection Center (CVCC)]. *Escherichia coli* DH5α, *Escherichia coli* BL21, *Escherichia coli* 83715, and *Lactobacillus acidophilus* ATCC SD5221 were used to determine host range of the isolated phages. All cultures were stored at -80°C in 18% glycerol.

### Media and Buffers

The 2^x^YT broth: Peptone 16 g/L, yeast extract powder 10 g/L, NaCl 5 g/L. The 2^x^YT-agar broth: All above mentioned ingredients with 1.5% agar for the bottom plate, 0.7% agar for the overlay. SM buffer: MgS0_4_⋅7H_2_O 2 g, NaCl 5.8 g, and 50 mL l mol/L Tris–HCI (pH 7.5) in 1 L. Tryptic soy broth (TSB): Tryptone 17 g, Soytone 3 g, glucose 2.5 g, NaCl 5 g, K_2_HPO_4_ 2.5 g in 1 L with adjusted pH 7.1–7.5. Buffered peptone water (BPW): Peptone 10 g/L, NaCl 5.0 g/L, Na_2_HPO_4_⋅12H_2_O 9.0 g/L, KH_2_PO_4_ 1.5 g/L in 1 L with adjusted pH 7.0–7.4.

### Isolation and Purification of Phages

Samples were collected from multiple environmental sources in Wuhan, Hubei-China including a wastewater treatment plant, sewage near the river, farm ditch near the lake, chicken and pigs’ feces. Samples were centrifuged at 10,000 ×*g* for 10 min to remove solid particles and bacteria were excluded using a 0.22 μm (Millipore, Ireland) sterile filter. As for chicken and pigs’ feces, samples were dissolved in 10 mL/g 2^x^YT media before centrifugation.

For enrichment, *Salmonella* strain ATCC 13076 were used as the host strain since this serovar has being the most frequent one in EU and United States (CDC, 2015). It was grown 8–10 h at 37°C in tryptic soy broth (TSB) to obtain pure bacterial cultures. Two hundred microliter overnight cultures were inoculated into 10 mL TSB and incubated at 37°C shaker at the speed of 160 rpm for 6–8 h to reach the exponential growth phase. 10 mL *Salmonella* cultures were mixed with 40 mL 2^x^YT media and 10 mL filtered sample with the ratio of 1:4:1(v/v/v) to amplify the collected phages. Amplified phages were isolated by centrifugation at 8000 ×*g* for 15 min and filtration using 0.22 μm sterile filter. Both large and small phage plaques were picked. To do so, dilution series of isolated phage samples were assessed on plates covered in a lawn of target bacteria. Individual plaques were picked and re-purified for three consecutive passages.

### Host Range of Phages LPSE1

The host range of bacteriophages was determined by spotting 4 μL of phage lysates (10^8^ PFU/mL) onto lawns of test strains. The plates that containing lawns of test strains were prepared with a mixture of 200 μL and 3 mL 0.7% agar for the overlay. The plates were incubated at 37°C, additionally pullorum was tested at 42°C also and bacterial lysis was recorded. A common scoring system for determining phage infectivity was applied as follows: +4 complete clearing; +3 clearing throughout but with a faintly hazy background; +2 substantial turbidity throughout the cleared zone; +1 a few individual plaques; 0 no clearing (Clokie and Kropinski, 2009; Akhtar et al., 2014). Further determination of the efficiency of plating was conducted using a previously described method and measured by expressing the phage titer of the susceptible strain relative to the phage titer of the reference strain (Akhtar et al., 2014).

### Evaluation of Phage Virulence

All bacterial strains were streaked from the frozen culture stock onto 2^x^YT-agar. Prior to experimental evaluation, each strain was grown by picking an isolated colony from 2^x^YT-agar plates and inoculating into 10 mL 2^x^YT broth and incubated at 37°C to obtain fresh overnight cultures. Bacterial overnight cultures (10^6^ CFU/mL) were inoculated 1000:1 with defined phage lysates and incubated at 37°C in a TS-2 type cyclotron oscillator at 160 rpm. Sample OD_600__nm_ values were measured every hour for a total duration of 9 h (Infinite M200 Pro, Tecan, Switzerland) (McLaughlin, 2007).

### Characterization of Selected Phages

#### pH Stability

For pH-stability testing, 100 μL phage samples (10^8^ PFU/mL) were mixed in a series of tubes containing sterile BPW of various pH values ranging from 2–13 (adjusted using NaOH or HCl) and incubated for 2 h at 37°C. Bacteriophage titers were determined using *Salmonella* Enteritidis ATCC13076 as the host on the double-layer agar plate (López-Cuevas et al., 2011). The rates of phage pH/thermal stability were calculated in the formula: Phage stability rate (%) = phage concentration (PFU/mL) under certain condition/initial phage concentration added (PFU/mL) × 100%.

#### Thermal Stability

For thermal-stability testing, 100 μL phage lysates (10^8^ PFU/mL) were mixed with 900 μL pre-heated sterile 2^x^YT medium. Samples were maintained in a water bath ranging from 30°C to 80°C for either 30 or 60 min. Bacteriophage titers were determined using *Salmonella* Enteritidis ATCC13076 as the host on the double-layer agar plate (López-Cuevas et al., 2011).

#### Morphology of LPSE1

Phage lysates (10^9^ PFU/mL) were re-suspended in SM buffer solution after ultra-centrifuging at 50,000 ×*g* for 2 h. Samples were placed in an ice bath for 1–2 h and prepared using negative staining method before applied to electron microscopical grids and negatively stained with 2%, pH 7.0 phosphotungstic acid (PTA). The preparations were allowed to dry in the chamber at room temperature and were then examined under transmission electron microscope (TEM) (Hitachi H-7000FA, Tokyo, Japan) and analyzed using software Digital Micrograph Demo 3.9.1.

#### DNA Analysis LPSE 1

The nucleic acid of the phage was extracted according to a previously described method (Clokie and Kropinski, 2009) using 10% SDS and proteinase K (10 mg/mL). H*ind*III and E*coR*V were chosen to used as the enzyme for restriction enzyme digestion. LPSE1 DNA was extracted and purified according to the previous literature (Clokie and Kropinski, 2009). The genome was sequenced on the HiSeq platform (Illumina, San Diego, CA, United States) using a paired-end library with a 151 bp read length. The sequences were assembled using Newbler (version 2.8) resulting in a unique contig. Putative coding DNA sequences (CDSs) were identified by Glimmer 3.0 (Delcher et al., 2007) and the length of open reading frames (ORFs) were set to more than 110 bp. Functional annotation of CDSs was performed by searching against nr protein database using BLASTP (Altschul et al., 1997). The complete genome sequence of *Salmonella* phages LPSE1 has been deposited in GenBank under the accession number KY379853.

#### Phage Replication Kinetics

The single-step growth curve was measured by using a previously described method (Pajunen et al., 2000; Sillankorva et al., 2008) with slight modifications. Briefly, *Salmonella* strains ATCC 13076 grown to mid-exponential phase (6–8 h) were harvested and adjusted to an OD_600__nm_ of 0.5. The bacterial suspension was inoculated with purified phage lysates to a multiplicity of infection (MOI) of 0.01, and phages were allowed to be adsorbed for 15 min at 37°C. The mixture was then centrifuged at 7,000 ×*g* for 2 min and the bacterial–phage pellet was suspended in an equal volume of fresh 2^x^YT medium. Thereafter, 50 μL of the suspension was added to 50 mL of pre-heated 2^x^YT culture and incubated at 37°C with constant shaking (160 rpm). Aliquots of 50 μL were removed every 10 min over a period of 120 min. Aliquots were centrifuged at 13,000 *g* for 30 s, and 20 μL of the supernatant was utilized to determine the phage titer using the double-layer agar plate method (López-Cuevas et al., 2011).

### Phage Stability

Phage stability was assessed by adding 100 μL bacteriophage (10^8^ PFU/mL) to 50 mL of nutrient broth to a final concentration of 10^5^ PFU/mL (as the phage titer in application part is 10^5^ PFU/mL) and incubated at 37°C with shaking at 100 rpm (Cairns et al., 2009). Samples were taken in 0 and 12 h and 1, 2, 4, 5, and 7 days and bacteriophages were enumerated as previously described.

### Application

#### Assays in Milk

Fresh skim milk was prepared using the skim milk powder from BD-Difco Company, United States and was sterilized according to manufacturer’s instructions. 100 μL phage lysates (10^6^ CFU/mL) were added to milk inoculated with 10 μL *Salmonella* Enteritidis ATCC 13076 at an MOI of 1 (10^7^ CFU/mL) or MOI of 100 (10^5^ CFU/mL). Equal volume of SM buffer was added to the milk in the control group. Samples were incubated at 4 or 28°C. After 0, 1, 2, 4, and 6 h of incubation, aliquots were drawn to determine viable bacterial counts (CFU/mL) and phage concentrations (PFU/mL). Recoverable bacteria were enumerated by serial plating. Phage concentration was assessed by centrifuging the aliquot at 11,000 ×*g* for 10 min and determining phage present in the supernatant.

#### Assays in Sausage

Packed pork sausages were cut into a particular size (diameter: 2 cm, thickness: 1 cm) using a sterile knife and were placed in a sterile petri dish. Sausage sample sterility was ensured by placing a section on a TSA plate. The sausage sections were covered with *Salmonella* Enteritidis ATCC 13076 and an MOI of 1 or 100 of phage lysate. Equal volume of SM buffer was added to the sausage in the control group. Samples were placed in the safety cabinet to dry for 15–20 min before adding the phage lysate and incubated at 4 or 28°C. Samples were taken after 0, 1, 2, 4, and 6 h of incubation, added the sausage into 5 mL sterile buffer solution and cleaned the samples for 15 min in 96 W using ultrasonic cleaner. Samples were extensively mixed to obtain a uniform homogenate before determining viable bacterial count (CFU/mL) and phage concentration (PFU/mL).

#### Assays in Lettuce

Lettuce was obtained from a local supermarket. Inner leaves were hand-cut into pieces using a sharp knife. Lettuce was cleaned with distilled water and alcohol swab for the initial surface decontamination. Lettuce leaves were cut into a 1.5 cm-diameter shreds. These leaves were sterilized under UV exposure for 20 min. Sterility was ensured by placing a leaf sample on a fresh TSA plate. Lettuce shred were covered with *Salmonella* Enteritidis ATCC 13076. Lettuce was left to dry for 60 min with a sterilize paper containing sterilized water to keep a stable relative humidity. Phage lysates were added onto the lettuce at an MOI of 1, 10, or 100. Equal volume of SM buffer solution was added in the control group. Aliquots were extracted after 0, 1, 2, 3, 4, and 5 h of incubation at 24°C and suspended in 2 mL sterile SM buffer solution. Suspended samples were homogenated with sterile bars and vortexed for 30 s. Viable bacteria counts (CFU/mL) were determined by serial dilution plate enumeration.

#### Statistical Analysis

All measurements were conducted in triplicate except pH and thermal stability and application of phage in different foods which were done in duplicates. The populations of microbes were evaluated as means of the biological replications. The one way ANOVA (SPSS Inc. IBM corporation) was used to determine the significance of the application of phages in different foods. Level of significance was defined at *P* ≤ 0.05.

## Results And Discussion

### Initial Screening and Host Range of LPSE1

**FIGURE 1:**
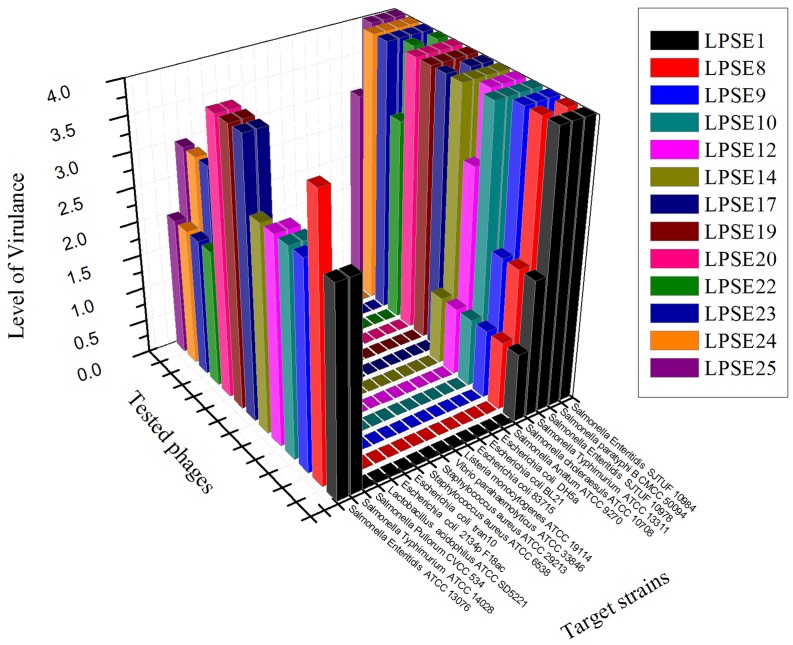
Host range of isolated phages against different strains. All experiments were conducted at 37°C (*Salmonella enterica* serovar pullorum was also tested at 42°C) with 10^8^ PFU/mL of phage. Scale: 4, Complete clearing; 3, clearing throughout but with faintly hazy background; 2, substantial turbidity throughout the cleared zone; 1, a few individual plaques; 0, no clearing - but a spot can be seen where the pipette tip touched the agar.

Thirty-five different phages were isolated from environmentally acquired samples including domestic sewage, municipal treatment plant, poultry and swine. The pooled feces samples are always of a great interest for amplification of lytic phages against different serovars of *Salmonella* (O’Flynn et al., 2006) and lytic phages from sewage and municipal waste are also helpful in isolation of strong phages (Carey-Smith et al., 2006). The greatest number of phages was isolated from domestic sewage (15) and poultry (10) sources. Phages isolated and amplified using *Salmonella* Enteritidis ATCC 13076 were denoted as LPSE1–LPSE35. The host range of amplified phages in this study was determined (**Figure 1**). Among the tested phages, LPSE17, LPSE19, and LPSE20 demonstrated the greatest lytic activity against the evaluated host strains. LPSE1, LPSE10, LPSE23, and LPSE25 also showed high lytic activity but it was bit lower than the aforementioned 3 phages. All others phages’ activity was weaker. Two of the serovars of *Salmonella* were found to be resistant against all tested phages i.e., *Salmonella enterica* serovar Pullorum and *Salmonella enterica* serovar Anatum. This resistance is probably due to strict range of the amplified phages or presence of some special phage resistance mechanism including receptors which don’t allow the phages to adsorb to host and fail to enter their nucleic acid (Labrie et al., 2010). Among all tested phages, no phage broke the boundary of genus showing that these had the potential to be used for effective control of *Salmonella* minimally disturbing other micro-flora present in food stuff. The strict host range is in accordance with the previous phages isolated by different researchers proving the phages to be a safe and well aimed candidate to be applied in different foods (Kang et al., 2013; Akhtar et al., 2014)

### Phage Virulence

**FIGURE 2:**
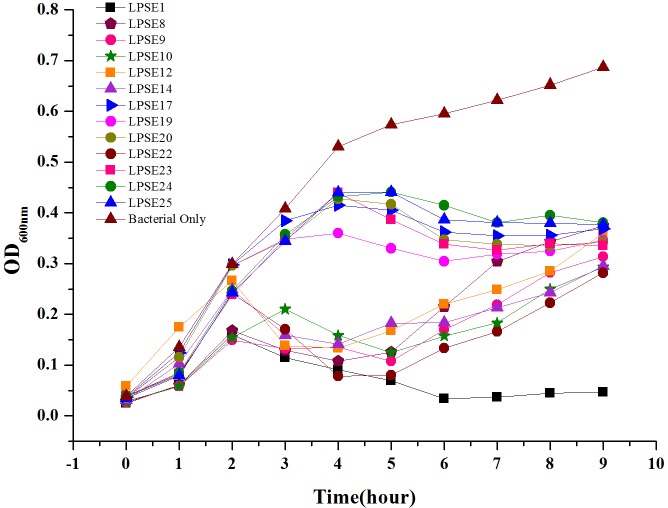
Lysis ability comparison of selected phages. All experiments were conducted at 37°C and samples were drawn after every 1 h. Data reported are means ± standard deviations of three independent trials.

All selected phages were tested against *Salmonella* Enteritidis ATCC 13076 to evaluate their virulence (**Figure 2**). All phages reduced *Salmonella* proliferation relative to the negative control. No significant change in virulence was observed amongst the phages for first 2 h post-inoculation. However, eight phages demonstrated enhanced virulence after 4.5 h post-inoculation. Only phage LPSE1 continued to suppress *Salmonella* Enteritidis ATCC 13076 proliferation up to 10 h post-inoculation whereas, all other collected phages permitted growth. The extended bacterial suppression afforded by LPSE1 indicates evasion of bacterial resistance, a major hurdle in phage therapy applications (García et al., 2008). Thus, phage LPSE1 was selected for further efficacy and stability analyses.

### Lytic Ability of LPSE1

**FIGURE 3:**
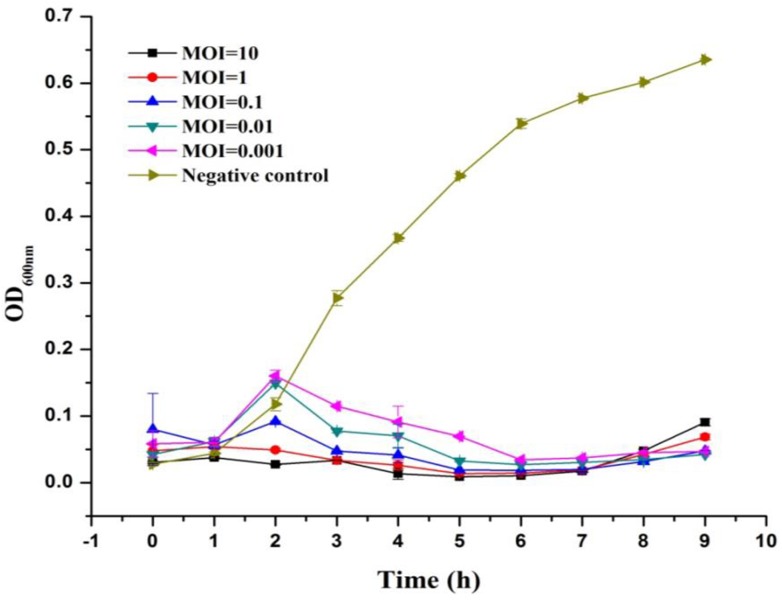
Lysis ability comparison of LPSE1 at different MOI. LPSE1 concentration dependent *Salmonella* Enteritidis ATCC 13076 antimicrobial efficacy was evaluated at an MOI of 0.001, 0.01, 0.1, 01, or 10 upon incubation at 37°C. Data reported are means ± standard deviations of three independent trials.

The lytic ability of phage LPSE1 upon infection of *Salmonella* Enteritidis ATCC 13076 was further established at various MOIs (**Figure 3**). The LPSE1 showed very vigorous lytic ability against its host regardless of the dose applied. Inoculation of an MOI of 0.001 was sufficient to prevent an appreciable change *Salmonella* could have gained relative to the initial inoculum after 9 h of incubation. To the contrary, significant increase was noted in the bacterial concentration ending up with OD_600__nm_ of 0.6 which was OD_600__nm_ 0.5 in the beginning.. In cultures applied with phage titers of MOI 0.1, MOI 0.01, and MOI 0.001, an initial rise of 0.05 to 0.1 OD_600__nm_ was observed after 2 h if incubation. However, this rise was dematerialized gradually by 6 h post-inoculation. From these results it is obvious that all of the amplified phages can constantly inhibit the growth of *Salmonella* at any applied concentration. The initial rise in *Salmonella* number is due to higher gap between concentration of phages and host. After some time, as infection and multiplication started and phage concentration was raised, *Salmonella* was easily controlled and decreased to very low number.

### Morphology of DNA Analysis LPSE 1

**FIGURE 4:**
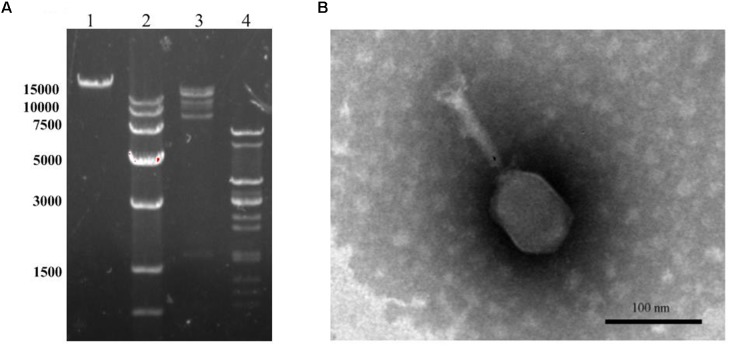
Morphology analyses of LPSE1. **(A)** Restriction analyses of LPSE1 genome; 1; Complete Genome of LPSE1, 2; Marker (size is shown in bp), 3; Nucleic Acid digested by H*ind*III, 4; Nucleic acid digested by E*coR*V. **(B)** Transmission Electron Microscope analysis of LPSE1; LPSE1 belongs to *Siphoviridae* family with 116.6 nm long, 16.6 nm wide tail and 70 nm wide head.

Transmission electron microscope image for LPSE1 was resolved (**Figure 4A**). LPSE1 has isometric head of 70 nm diameter and a long non-contractile tail of 116.6 nm long and 6.6 nm wide. Extracted DNA of LPSE1 was incubated in presence of ds-DNA restriction enzymes: H*ind*III and E*coR*V separately which generated multiple bands on the resolved gel and confirmed the nucleic acid as ds-DNA. The genome size of LPSE1 was 41.2 kb which is in the range of characteristic size, 17–498, of tailed phages (Clokie and Kropinski, 2009) (**Figure 4B**). The LPSE1 genome contains 41,854 bp and has a GC content of 49.83%. The genome contains 61 predicted genes, with an average gene length of approximately 596 bp. Sixteen of the genes are rightward oriented, while 45 are leftward oriented. Out of the putative CDSs, 59 (96.7%) have predicted proteins with similar counterparts in other genomes, and 2 (3.3%) have no substantial similarity with known proteins. Based upon predicted annotations, this phage genome contains structural, replication, and lysis factors (Supplementary Table 1). These characteristics show that LPSE1 belongs to *Siphoviridae* family of bacteriophages. Other members of family *Siphoviridae* were reported to impart a 50 nm-78.8 nm wide non-capsulated head group and a non-contractile tail of 100 nm-167.7 nm long and 4 nm-9 nm wide (Carey-Smith et al., 2006; O’Flynn et al., 2006; Pao et al., 2006; Kocharunchitt et al., 2009; Bao et al., 2011; Lu et al., 2015). The members of the *Siphoviridae* tend to be present in water and are somatic phages with can infect host bacteria at any time and can also attach to host even it is dead (Grabow, 2001). In the previous experiments, researchers have successfully controlled different serovars of *Salmonella* in broiler chicken using phages belonging to *Siphoviridae* family (Atterbury et al., 2003; Kang et al., 2013), characteristically controlled *Salmonella* Enteritidis and Typhimurium in different vegetable seeds (Pao et al., 2006) and in seed sprouts (Kocharunchitt et al., 2009). These evidences indicate that LPSE1 is a promising candidate in application against pathogenic *Salmonella* in different foods.

### pH and Thermal Tolerance

**FIGURE 5:**
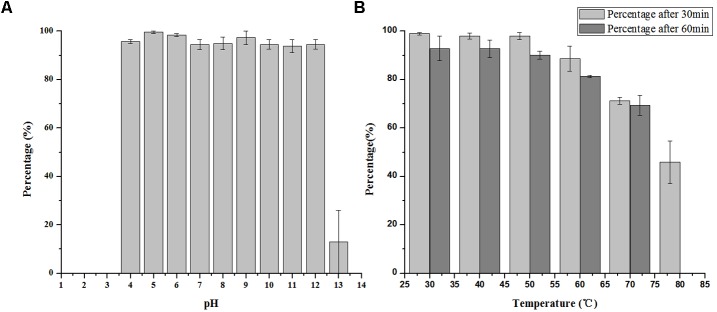
Stability of LPSE1 on different pH and temperatures. **(A)** pH stability of LPSE1; **(B)** thermal stability of LPSE1; All the experiments are conducted on 37°C (Otherwise mentioned). Data reported are means ± standard deviations of three independent trials. Error bars show the deviation in the values.

LPSE1 is very stable bacteriophage showing pH resistance ranging from 4–12 after 2 h (**Figure 5A**). Recoverable LPSE1 titers remained active throughout pH 4–12. LPSE1 titers declined when applied to pH extremes of either less than 3 or greater than 13. Phage LPSE1 also exhibited a high degree of thermal tolerance with active titer as high as 70°C (**Figure 5B**). When heated at 80°C for 30 min LPSE1 titers declined. No viable LPSE1 phage was detected upon heating at 80°C for 60 min. The pH and thermal tolerance of LPSE1 was better than previously reported phages having 4–10 pH and 60°C (Bao et al., 2011), 4–11 pH and 70°C (Bao et al., 2015) and 4–12 pH and 60°C reported by (Lu et al., 2015). Phage application with better range can give a broader window of application in food stuff with lower or higher pH and with or without thermal treatment. Heat and pH resistant phage application adds an advantage in treatment against pathogens as only heat or pH can’t fully kill the pathogens. This argument is supported by the data given (Uyttendaele et al., 2009; Lambertz et al., 2012) showing existence of pathogens even after heat treatment to meat products. Some studies show prevalence of pathogens lower than 100 CFU/g (Modzelewska-Kapituła and Maj-Sobotka, 2013) but some are in contradiction to this and show even higher than 100 CFU/g (Garrido et al., 2009).

### Phage Stability

**FIGURE 6:**
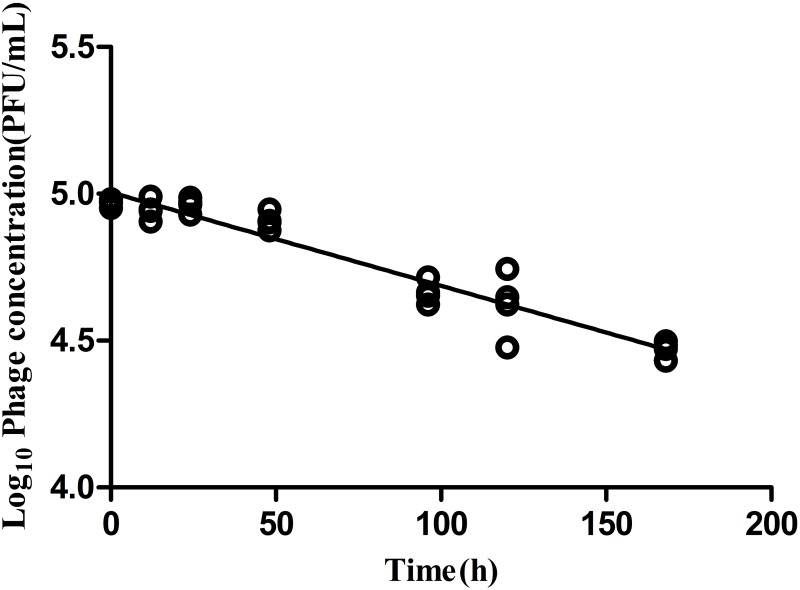
Analysis of phage stability in the absence of host. Culture was inoculated at 37°C in shaking incubator with 5.0 log_10_ and decay was recorded for 168 h.

Phage stability in medium lacking a permissible host is a critical parameter to evaluate bio-control application efficacy. In the absence of a bacterial host, LPSE1 exhibited gradual and constant deterioration over a period of 168 h of incubation at 37°C (**Figure 6**). After 168 h of incubation, there was a 0.5 log_10_ reduction of LPSE1 phage titer. The calculated deterioration rate was 2.9 × 10^-3^ log_10_ per hour exhibiting good stability of LPSE1. This results are in accord with results presented by (Cairns et al., 2009) showing a phage decay rate of 1.06 × 10^-2^ log_10_ per hour. In previous studies, phage stability is measured in samples showing that phage is stable for 24 h and then it start deteriorating significantly in Chinese cabbage and chicken breast but phages showed better stability in milk (Bao et al., 2015) but this data was recorded as long as 72 h. Another study on stability of phages recovered and applied to different food matrices show phages with different stability at different temperatures (Robeson et al., 2014). In the presence of host, phage shows better stability as proven by (Guenther et al., 2012) by spiking samples with host and phage at the same time and recorded phage stability as long as 6 days in different food stuff with no considerable increase or decrease.

### One Step Growth Curve

**FIGURE 7:**
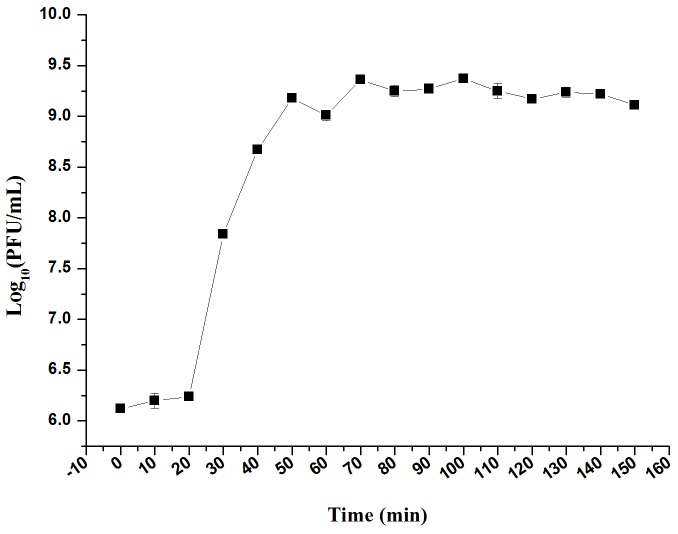
One-step growth curve of LPSE1. Phage was incubated at 37°C, samples were drawn every 10 min. Data reported are means ± standard deviations of three independent trials. Error bars show the deviation in the values.

The infection dynamics of phage LPSE1 were examined (**Figure 7**). The majority of LPSE1 phage particles infected host *Salmonella* Enteritidis ATCC 13076 within 20 min post-inoculation. A very long initial period is probably due to less approach of LPSE1 to the host but the higher number of PFU per cell was very high in first burst as compared to second one which was defined by presence of higher number of available host cells which decreased slowly in medium. Following a short latent period, LPSE1 phage particles were generated exponentially. The average burst was calculated to be approximately 94 PFU/CFU.

### Application of Phage LPSE1 in Controlling Foodborne *Salmonella enterica*

#### Milk

**FIGURE 8:**
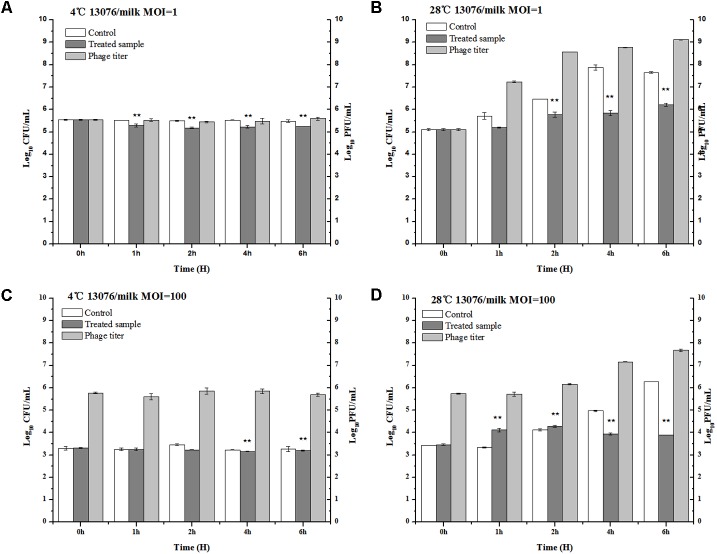
Application of LPSE1 in milk. **(A)** LPSE1 applied at 4°C using MOI of 1; **(B)** LPSE1 applied at 28°C using MOI of 1; **(C)** LPSE1 applied at 4°C using MOI of 100; **(D)** LPSE1 applied at 28°C using MOI of 100, Values are indicated as means of biological replicates. ^∗∗^Highly Significant *P*-value < 0.05 (Tukey’s B).

Milk, a broadly available and highly consumed product, is collected and stored in various forms and conditions. The influence of bacteriophage LPSE1 against *Salmonella* Enteritidis ATCC 13076 was assessed at both 4°C, lower than average refrigeration temperature, and 28°C, average the room storage temperature (Laguerre et al., 2002) (**Figure 8**). When applied at an MOI of 1 or 100 at 4°C, LPSE1 did not confer an appreciable change upon viable *Salmonella* CFUs or phage replication PFUs statistics. The low temperature is one of the reasons of the low virulence of phage as low temperature hinder the growth of microbes and phages are dependent on the multiplication of its host. However, when applied at an MOI of 1 or 100 at 28°C, the LPSE1 titer rose over the course of 6 h incubation to 9.12 log_10_ PFU/mL or 7.67 log_10_ PFU/mL, respectively. Administration of LPSE1 reduced recoverable *Salmonella* by 1.44 log_10_ CFU/mL at an MOI of 1 or 2.37 log_10_ CFU/mL at an MOI of 100, relative to the phage-excluded control. Despite a significant reduction in detectable *Salmonella* following LPSE1 treatment relative to the non-treated control at 28°C, in the total number of recoverable *Salmonella* increased throughout the experimental duration. When administered at an MOI of 1, LPSE1 permitted an overall increase in *Salmonella* concentration 1.22 log_10_ CFU/mL; whereas, the non-treated control was enhanced by 2.5 log_10_ CFU/mL. More strikingly, when introduced at an MOI of 100, LPSE1 largely suppressed *Salmonella* proliferation resulting in a 0.44 log_10_ CFU/mL increase. In the absence of LPSE1 treatment, *Salmonella* was multiplied by 2.81 log_10_ CFU/mL. The reason behind the increase in *Salmonella* count upon incubation in milk at 28°C is may be attributed to favorable replication conditions. This argument is in accordance with a related study in which incubation of chocolate milk were artificially spiked with 10^3^ CFU/mL *Salmonella* Typhimurium. No detectable bacterial multiplication was observed for up to 6 days upon incubation at 8°C. However, upon incubation at 15°C, *Salmonella* Typhimurium rapidly multiplied after 48 h post-inoculation (Guenther et al., 2012). On the other hand, storage temperature may also influence phages stability or replication kinetics. As detailed in another study, multiple distinct phages were applied in milk against *Salmonella* at 4 and 25°C, resulting in variable trends (Bao et al., 2015). Alternate factors present in milk, such as immune components or inhibitory proteins, may also hinder the efficacy of bacteriophages from gaining access to host bacteria (O’Flaherty et al., 2005; Gill et al., 2006).

#### Sausage

**FIGURE 9:**
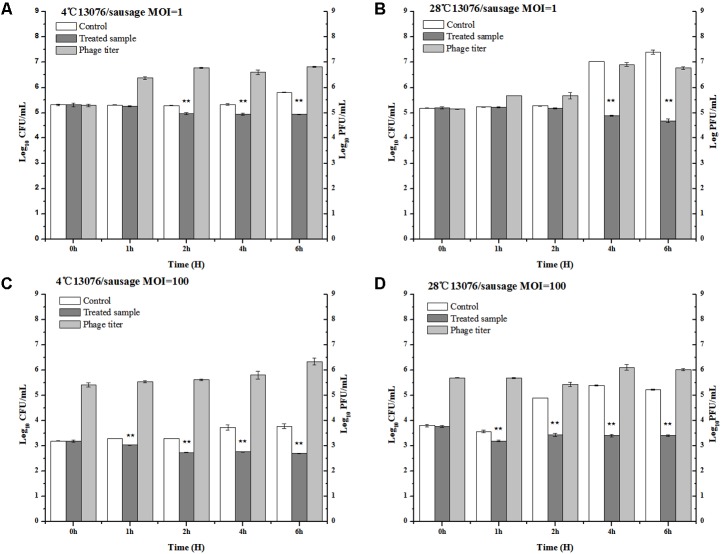
Application of LPSE1 in sausage. **(A)** LPSE1 applied at 4°C using MOI of 1; **(B)** LPSE1 applied at 28°C using MOI of 1; **(C)** LPSE1 applied at 4°C using MOI of 100; **(D)** LPSE1 applied at 28°C using MOI of 100, Values are indicated as means of biological replicates. ^∗∗^Highly Significant *P*-value < 0.05 (Tukey’s B).

As a versatile meat product comprised of various ingredients potentially contaminated with foodborne pathogens, sausages are of special consideration in regards to safe preparation. The anti-*Salmonella* efficacy of phage LPSE1 was assessed in pork sausage (**Figure 9**). At MOI of 1, application of LPSE1 at 4°C decreased the number of *Salmonella* from 5.32 to 4.94 log_10_ CFU/mL as opposed to an increase to 5.81 log_10_ CFU/mL observed for the non-treated control. This reduction in *Salmonella* corresponded with an increase of LPSE1 phage titer from 5.32 to 6.83 log_10_ PFU/mL. Application of LPSE1 at MOI of 100 at 4°C also resulted in reduced viable *Salmonella*. With an initial concentration of 3.2 log_10_ CFU/mL, recoverable *Salmonella* decreased to 2.71 CFU/mL upon LPSE1 administration; whereas, viable *Salmonella* increased to 3.78 CFU/mL in the absence of phage. The LPSE1 titer also increased from 5.2 log_10_ to 6.34 log_10_ PFU/mL when introduced at an MOI of 100 at 4°C. Comparable results were observed upon administration of LPSE1 on sausage at 28°C. Introduction of LPSE1 at an MOI of 1 reduced viable *Salmonella* from 5.2 to 4.68 log_10_ CFU/mL. To the contrary, the non-treated control demonstrated *Salmonella* proliferation up to 7.4 log_10_ CFU/mL. The LPSE1 phage titer was multiplied from 5.2 to 6.77 log_10_ PFU/mL. Administration of LPSE1 at an MOI of 100 decreased *Salmonella* colony forming units from 3.8 to 3.42 log_10_ CFU/mL. Once again, the non-treated control supported *Salmonella* growth to a final concentration of 5.32 log_10_ CFU/mL and a phage titer increase to 6.02 log_10_. Taken together, LPSE1 application on sausage demonstrated a constant overall reduction in viable *Salmonella* at both 4 and 28°C. The anti-*Salmonella* efficacy of LPSE1 demonstrated superior performance compared to a related study assessing extended application of phage at 18°C on sausage. In this study, a decreasing trend when incubated for at least 3 days and firstly increasing for 6 days and then gradually decreasing trend when applied on barbeque sausage. This took even longer time when same incubation was done at 4°C and it took 10 days to lower *Salmonella* count as maximum as 1.6 log_10_ CFU/mL (Galarce et al., 2016). Almost same trend was found when *Salmonella* was applied with different variants of phages (Whichard et al., 2003). The high degree of LPSE1 efficacy may be attributed to robust stability as previously evaluated (**Figures 5**, **6**). In addition to this, bacteriophage was applied and *Salmonella* elimination was recorded after 24 h to non-detectable limit in hot dogs and sliced turkey but after very small reduction was noted in mixed sea food (Guenther et al., 2012). *Salmonella* on chicken skin was gave about 3 log_10_ reduction when incubated for 24 h (Kang et al., 2013) more than 1.3 log_10_ reduction after 7 h (Spricigo et al., 2013).

#### Lettuce

**FIGURE 10:**
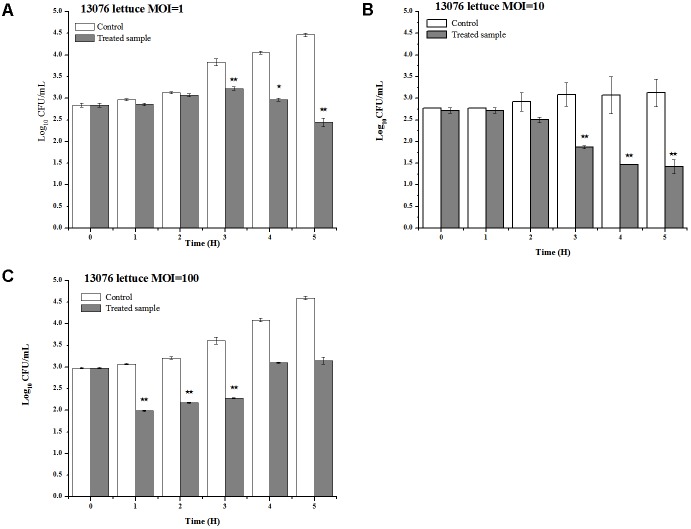
Application of LPSE1 on lettuce. **(A)** LPSE1 applied at MOI of 1; **(B)** LPSE1 applied at MOI of 10; **(C)** LPSE1 applied at MOI of 100; Values are indicated as mean of biological replicates. ^∗^Significant; ^∗∗^Highly Significant *P*-value < 0.05 (Tukey’s B).

Vegetables, including lettuce, are notoriously implicated to harbor foodborne pathogens (Spricigo et al., 2013). LPSE1 applied on lettuce leaves produced pragmatic results as shown in **Figure 10**. Relative to the non-treated controls, the viable *Salmonella* counts were reduced upon administration of LPSE1 at an MOI of 1, 10, and 100. Upon LPSE1 inoculation at an MOI of 1, the *Salmonella* count decreased to 2.4 log_10_ CFU/mL; whereas, viable *Salmonella* increased to 4.47 log_10_ CFU/mL in the untreated control. Similarly, at MOI of 10, recoverable *Salmonella* decreased to 1.42 log_10_ CFU/mL. The non-treated sample resulted in an increase in *Salmonella* count from the initial 2.77 log_10_ CFU/mL to 3.12 log_10_ CFU/mL. Administration at an MOI of 100, revealed a different trend. In the untreated control, the *Salmonella* count was raised to 4.6 log_10_ from the initial 2.97 log_10_ CFU/ml inoculum. However, upon introduction of LPSE1 at an MOI of 100, the *Salmonella* count decreased abruptly after 1 h. Thereafter, the recoverable *Salmonella* increased gradually to 3.15 log_10_ CFU/mL, a value slightly higher thanthe initial concentration. These results are in agreement with independent observations showing a 3 log_10_ reduction when a distinct phage was applied against *Salmonella* on Chinese cabbage (Bao et al., 2015), 1.7 log_10_ reduction on lettuce (Spricigo et al., 2013), 1.37 log_10_ on mustard and a 0.55 log_10_ reduction on broccoli (Pao et al., 2006).

## Conclusion

This study details the environmental isolation of bacteriophages shown to be efficacious against a broad range of human pathogenic *Salmonella* serotypes. Phage LPSE1, which demonstrated prolonged *in vitro* suppression of *Salmonella* Enteritidis ATCC 13076, was further characterized. LPSE1 exhibited robust pH and thermal stability as well as rapid anti-*Salmonella* lytic potential. Application of LPSE1 phage upon milk, sausage, and lettuce contaminated with *Salmonella* Enteritidis, reduced recoverable pathogen relative to the non-treated controls. Thus, these findings establish phage LPSE1 as an effective *Salmonella* bio-control agent in various ready to eat (RTE) food preparations. Additional studies are necessary to further detail and refine phage-mediated foodborne pathogen control applications.

## Author Contributions

JqL and XW planned the experiments. CH, JS, and JL did the experiments and analyses. SV wrote the manuscript. JqL, YZ, SW, MM, and HA revised the manuscript.

## Conflict of Interest Statement

The authors declare that the research was conducted in the absence of any commercial or financial relationships that could be construed as a potential conflict of interest.
