# Supplementary material for: Isolation, Characterization, and Application of Bacteriophage LPSE1 Against Salmonella enterica in Ready to Eat (RTE) Foods
Source: Front Microbiol. 2018 May 23;9:1046. doi: 10.3389/fmicb.2018.01046 (PMC5982681; doi:10.3389/fmicb.2018.01046)
Supplement: Supplementary file 1 [file Table_1.DOC]

Supplementary Material

**Isolation, characterization, and application of bacteriophage LPSE1 against *Salmonella enterica* in ready to eat (RTE) foods**

Chenxi Huang^a, #^, Virk Muhammad Safiullah ^a, #^, Jianchun Shi^a^, Yang Zhou^c, d^, Stephan P. Willias ^c^, Mohamed Khairy Morsy^b^, Hazem Elewa Abdelnabby^b^, Jie Liu^e^, Xiaohong Wang^a*^, Jinquan Li^a*^

Bio-Medical Center, Key Laboratory of Environment Correlative Dietology, State Key Laboratory of Agricultural Microbiology, College of Food Science and Technology, Huazhong Agricultural University, Wuhan, Hubei, People’s Republic of China^a^; Department of Food Science, Faculty of Agriculture, Benha University, Qaluobia, Egypt^b^; Department of Infectious Diseases and Pathology, University of Florida, Gainesville, Florida, USA^c^; College of Fisheries, Huazhong Agricultural University, Wuhan, Hubei, People’s Republic of China^d^; College of Medicine, Hebei University of Engineering, Handan, Hebei Province, P. R. China^e^;

*** Correspondence:** Corresponding Author: Xiaohong Wang, Jinquan Li

E-mail: [wxh@mail.hzau.edu.cn](mailto:wxh@mail.hzau.edu.cn); [lijinquan2007@gmail.com](mailto:lijinquan2007@gmail.com);

#C.H. and V.M.S. contributed equally to this work.

**Table 1.** Functional annotation of LPSE1 CDSs using BLASTP

| **Query_name** | **Query_description** | **Hit_description** |
| --- | --- | --- |
| phage_orf00001 | 450-1193 -3 length=247 | gp39 [Sodalis phage SO-1] gi|258619878|gb|ACV84131.1| gp39 [Sodalis phage SO-1] |
| phage_orf00003 | 2684-3310 -2 length=208 | hypothetical protein SPSV3_gp11 [Salmonella phage SETP3] gi|125631937|gb|ABN47340.1| hypothetical protein [Salmonella phage SETP3] |
| phage_orf00004 | 3392-4666 -2 length=424 | hypothetical protein [Salmonella phage SS3e] gi|57472381|gb|AAW51243.1| hypothetical protein [Salmonella phage SS3e] |
| phage_orf00006 | 4708-5100 -1 length=130 | hypothetical protein SPSV3_gp13 [Salmonella phage SETP3] gi|125631939|gb|ABN47342.1| hypothetical protein [Salmonella phage SETP3] |
| phage_orf00008 | 5377-5595 +1 length=72 | hypothetical protein SPSV3_gp14 [Salmonella phage SETP3] gi|375267233|ref|YP_005098108.1| hypothetical protein [Salmonella phage SE2] gi|423261888|ref|YP_007010495.1| putative DNA-binding protein [Salmonella phage vB_SenS-Ent1] gi|125631940|gb|ABN47343.1| hypothetical protein [Salmonella phage SETP3] gi|126015308|gb|ABN70685.1| hypothetical protein [Salmonella phage SETP5] gi|126015310|gb|ABN70686.1| hypothetical protein [Salmonella phage SETP12] gi|371767634|gb|AEX56151.1| hypothetical protein [Salmonella phage SE2] gi|387155605|emb|CCG55224.1| putative DNA-binding protein [Salmonella phage vB_SenS-Ent1] |
| phage_orf00010 | 5610-7796 -3 length=728 | hypothetical protein [Salmonella phage ST4] |
| phage_orf00012 | 7853-7957 -2 length=34 | putative uvsX-like protein [Salmonella phage vB_SenS-Ent1] gi|387155607|emb|CCG55226.1| putative uvsX-like protein [Salmonella phage vB_SenS-Ent1] |
| phage_orf00013 | 8083-8253 -1 length=56 | hypothetical protein SPSV3_gp16 [Salmonella phage SETP3] gi|125631942|gb|ABN47345.1| hypothetical protein [Salmonella phage SETP3] |
| phage_orf00015 | 9064-9273 +1 length=69 | hypothetical protein [Escherichia coli] gi|345340083|gb|EGW72504.1| hypothetical protein ECSTECC16502_1335 [Escherichia coli STEC_C165-02] |
| phage_orf00016 | 9394-9558 +1 length=54 | hypothetical protein SPSV3_gp18 [Salmonella phage SETP3] gi|125631944|gb|ABN47347.1| hypothetical protein [Salmonella phage SETP3] |
| phage_orf00018 | 9734-10084 +2 length=116 | hypothetical protein [Salmonella phage SE2] gi|371767639|gb|AEX56156.1| hypothetical protein [Salmonella phage SE2] gi|398255764|gb|AFO70785.1| hypothetical protein [Salmonella phage ST4] |
| phage_orf00019 | 10066-10284 +1 length=72 | hypothetical protein SPSV3_gp19 [Salmonella phage SETP3] gi|125631945|gb|ABN47348.1| hypothetical protein [Salmonella phage SETP3] |
| phage_orf00021 | 10287-10652 +3 length=121 | hypothetical protein [Salmonella phage SS3e] gi|57472369|gb|AAW51231.1| hypothetical protein [Salmonella phage SS3e] gi|397140416|gb|AFO12354.1| hypothetical protein [Salmonella phage wksl3] |
| phage_orf00022 | 10658-11092 +2 length=144 | hypothetical protein [Salmonella phage SE2] gi|371767642|gb|AEX56159.1| hypothetical protein [Salmonella phage SE2] |
| phage_orf00023 | 11171-11452 +2 length=93 | hypothetical protein SPSV3_gp21 [Salmonella phage SETP3] gi|125631947|gb|ABN47350.1| hypothetical protein [Salmonella phage SETP3] |
| phage_orf00024 | 11722-12210 +1 length=162 | lysozyme [Salmonella phage SETP3] gi|125631949|gb|ABN47352.1| lysozyme [Salmonella phage SETP3] gi|126015312|gb|ABN70687.1| lysozyme [Salmonella phage SETP5] gi|126015314|gb|ABN70688.1| lysozyme [Salmonella phage SETP12] |
| phage_orf00025 | 12396-12581 +3 length=61 | hypothetical protein [Salmonella phage SS3e] gi|57472365|gb|AAW51227.1| hypothetical protein [Salmonella phage SS3e] gi|397140411|gb|AFO12349.1| hypothetical protein [Salmonella phage wksl3] |
| phage_orf00027 | 12578-12736 +2 length=52 | hypothetical protein SPSV3_gp25 [Salmonella phage SETP3] gi|125631951|gb|ABN47354.1| hypothetical protein [Salmonella phage SETP3] |
| phage_orf00028 | 12733-12888 +1 length=51 | hypothetical protein [Salmonella phage vB_SenS-Ent1] gi|387155618|emb|CCG55237.1| hypothetical protein [Salmonella phage vB_SenS-Ent1] |
| phage_orf00029 | 12885-13241 +3 length=118 | putative NinH-like protein [Salmonella phage vB_SenS-Ent1] gi|387155619|emb|CCG55238.1| putative NinH-like protein [Salmonella phage vB_SenS-Ent1] |
| phage_orf00030 | 13259-13537 +2 length=92 | hypothetical protein [Salmonella phage vB_SenS-Ent1] gi|387155620|emb|CCG55239.1| hypothetical protein [Salmonella phage vB_SenS-Ent1] |
| phage_orf00032 | 13553-13735 +2 length=60 | hypothetical protein [Salmonella phage SE2] gi|371767650|gb|AEX56167.1| hypothetical protein [Salmonella phage SE2] |
| phage_orf00034 | 13796-13927 +2 length=43 | hypothetical protein [Salmonella phage wksl3] |
| phage_orf00035 | 13956-14462 +3 length=168 | hypothetical protein [Salmonella phage SE2] gi|371767652|gb|AEX56169.1| hypothetical protein [Salmonella phage SE2] |
| phage_orf00036 | 14452-15723 +1 length=423 | hypothetical protein [Salmonella phage ST4] |
| phage_orf00037 | 15736-17211 +1 length=491 | structural protein [Salmonella phage SS3e] gi|57472356|gb|AAW51218.1| structural protein [Salmonella phage SS3e] gi|397140400|gb|AFO12338.1| putative structural protein [Salmonella phage wksl3] |
| phage_orf00038 | 17242-17895 -1 length=217 | amidase [Salmonella phage wksl3] |
| phage_orf00040 | 18074-19933 +2 length=619 | hypothetical protein [Salmonella phage ST4] |
| phage_orf00041 | 19936-20394 +1 length=152 | putative head decoration protein [Salmonella phage vB_SenS-Ent1] gi|387155569|emb|CCG55188.1| putative head decoration protein [Salmonella phage vB_SenS-Ent1] |
| phage_orf00043 | 20712-21098 +3 length=128 | hypothetical protein [Salmonella phage vB_SenS-Ent1] gi|387155570|emb|CCG55189.1| hypothetical protein [Salmonella phage vB_SenS-Ent1] |
| phage_orf00044 | 21082-21207 +1 length=41 | hypothetical protein [Salmonella phage vB_SenS-Ent1] gi|387155571|emb|CCG55190.1| hypothetical protein [Salmonella phage vB_SenS-Ent1] |
| phage_orf00045 | 21292-21993 +1 length=233 | hypothetical protein [Salmonella phage SS3e] gi|57472351|gb|AAW51213.1| hypothetical protein [Salmonella phage SS3e] |
| phage_orf00046 | 21997-23046 +1 length=349 | hypothetical protein [Salmonella phage SE2] gi|371767661|gb|AEX56178.1| hypothetical protein [Salmonella phage SE2] |
| phage_orf00047 | 23107-23391 +1 length=94 | putative head protein [Salmonella phage wksl3] |
| phage_orf00048 | 23403-23753 +3 length=116 | hypothetical protein [Salmonella phage SS3e] gi|57472348|gb|AAW51210.1| hypothetical protein [Salmonella phage SS3e] |
| phage_orf00049 | 23790-23978 +3 length=62 | hypothetical protein [Salmonella phage vB_SenS-Ent1] gi|387155576|emb|CCG55195.1| hypothetical protein [Salmonella phage vB_SenS-Ent1] gi|485724999|gb|AGK86500.1| hypothetical protein [Salmonella phage L13] |
| phage_orf00050 | 23982-24491 +3 length=169 | hypothetical protein [Salmonella phage SS3e] gi|57472346|gb|AAW51208.1| hypothetical protein [Salmonella phage SS3e] |
| phage_orf00051 | 24494-25099 +2 length=201 | hypothetical protein [Salmonella phage wksl3] |
| phage_orf00053 | 25099-25458 +1 length=119 | putative tail protein [Salmonella phage wksl3] |
| phage_orf00054 | 25455-25850 +3 length=131 | hypothetical protein SPSV3_gp44 [Salmonella phage SETP3] gi|423261863|ref|YP_007010470.1| putative structural protein [Salmonella phage vB_SenS-Ent1] gi|125631970|gb|ABN47373.1| hypothetical protein [Salmonella phage SETP3] gi|387155580|emb|CCG55199.1| putative structural protein [Salmonella phage vB_SenS-Ent1] |
| phage_orf00055 | 25850-26269 +2 length=139 | hypothetical protein SPSV3_gp45 [Salmonella phage SETP3] gi|423261864|ref|YP_007010471.1| hypothetical protein [Salmonella phage vB_SenS-Ent1] gi|125631971|gb|ABN47374.1| hypothetical protein [Salmonella phage SETP3] gi|387155581|emb|CCG55200.1| hypothetical protein [Salmonella phage vB_SenS-Ent1] gi|397140450|gb|AFO12388.1| hypothetical protein [Salmonella phage wksl3] |
| phage_orf00056 | 26269-27438 +1 length=389 | putative tail protein [Salmonella phage wksl3] |
| phage_orf00057 | 27467-27811 -2 length=114 | putative DNA-binding protein [Salmonella phage SETP3] gi|125631973|gb|ABN47376.1| putative DNA-binding protein [Salmonella phage SETP3] |
| phage_orf00059 | 27855-28502 +3 length=215 | hypothetical protein [Salmonella phage SE2] gi|371767672|gb|AEX56189.1| hypothetical protein [Salmonella phage SE2] |
| phage_orf00061 | 28477-29607 -1 length=376 | hypothetical protein [Salmonella phage L13] |
| phage_orf00062 | 29670-29849 -3 length=59 | hypothetical protein SPSV3_gp50 [Salmonella phage SETP3] gi|423261870|ref|YP_007010477.1| putative imm immunity to superinfection membrane protein [Salmonella phage vB_SenS-Ent1] gi|125631976|gb|ABN47379.1| hypothetical protein [Salmonella phage SETP3] gi|387155587|emb|CCG55206.1| putative imm immunity to superinfection membrane protein [Salmonella phage vB_SenS-Ent1] gi|397140444|gb|AFO12382.1| hypothetical protein [Salmonella phage wksl3] |
| phage_orf00064 | 30019-30435 +1 length=138 | hypothetical protein [Salmonella phage wksl3] |
| phage_orf00065 | 30441-30800 +3 length=119 | hypothetical protein [Salmonella phage SS3e] gi|71801579|gb|AAZ41743.1| hypothetical protein [Salmonella phage SS3e] |
| phage_orf00066 | 30793-33126 +1 length=777 | putative tape measure protein [Salmonella phage vB_SenS-Ent1] gi|387155590|emb|CCG55209.1| putative tape measure protein [Salmonella phage vB_SenS-Ent1] |
| phage_orf00068 | 33185-33628 +2 length=147 | hypothetical protein [Salmonella phage wksl3] |
| phage_orf00069 | 33625-34140 +1 length=171 | hypothetical protein [Salmonella phage wksl3] |
| phage_orf00070 | 34137-34502 +3 length=121 | hypothetical protein [Salmonella phage vB_SenS-Ent1] gi|387155593|emb|CCG55212.1| hypothetical protein [Salmonella phage vB_SenS-Ent1] gi|485724983|gb|AGK86484.1| hypothetical protein [Salmonella phage L13] |
| phage_orf00071 | 34493-37051 +2 length=852 | hypothetical protein [Salmonella phage L13] |
| phage_orf00072 | 37064-39094 +2 length=676 | unnamed protein product [Salmonella phage SFP10] gi|345451193|gb|AEN94257.1| phage tailspike protein [Salmonella phage SFP10] |
| phage_orf00073 | 39206-39367 -2 length=53 | hypothetical protein [Salmonella phage L13] |
| phage_orf00074 | 39364-40704 -1 length=446 | putative helicase [Escherichia phage K1-ind(3)] gi|282547318|gb|ADA82375.1| putative helicase [Escherichia phage K1-ind(1)] gi|282547370|gb|ADA82426.1| putative helicase [Escherichia phage K1-ind(2)] |
| phage_orf00075 | 40785-40976 -3 length=63 | hypothetical protein [Salmonella phage SS3e] gi|375267224|ref|YP_005098099.1| hypothetical protein [Salmonella phage SE2] gi|57472388|gb|AAW51250.1| hypothetical protein [Salmonella phage SS3e] gi|371767625|gb|AEX56142.1| hypothetical protein [Salmonella phage SE2] |
| phage_orf00076 | 41008-41295 -1 length=95 | putative restriction endonuclease [Salmonella phage vB_SenS-Ent1] gi|387155599|emb|CCG55218.1| putative restriction endonuclease [Salmonella phage vB_SenS-Ent1] |
| phage_orf00077 | 41382-41735 -3 length=117 | DNA polymerase [Salmonella phage SE2] gi|371767627|gb|AEX56144.1| DNA polymerase [Salmonella phage SE2] |
